# UV-B Irradiation Effect on Microalgae Performance in the Remediation of Effluent Derived from the Cigarette Butt Cleaning Process

**DOI:** 10.3390/plants11182356

**Published:** 2022-09-09

**Authors:** Thais Huarancca Reyes, Lorenzo Mariotti, Carolina Chiellini, Lorenzo Guglielminetti, Gustavo Graciano Fonseca

**Affiliations:** 1 Faculty of Exact Sciences and Technology, Federal University of Grande Dourados, Dourados 79804-970, MS, Brazil; 2 Department of Agriculture, Food and Environment, University of Pisa, 56124 Pisa, Italy; 3 Centro di Ricerche Agro-Ambientali “E. Avanzi”, University of Pisa, 56122 Pisa, Italy; 4 Institute of Agricultural Biology and Biotechnology, Italian National Research Council, 56124 Pisa, Italy; 5 Faculty of Natural Resource Sciences, School of Business and Science, University of Akureyri, 600 Akureyri, Iceland

**Keywords:** *Chlorella sorokiniana*, flavonoids, phenolic compounds, photosynthetic pigments, non-enzymatic antioxidants, nicotine, nicotyrine, ultraviolet light

## Abstract

In this study, the potential of ultraviolet B (UV-B) radiation to alleviate the effects of pollutants in cigarette butt wastewater (CBW) was investigated using different *Chlorella sorokiniana* strains (F4, R1 and LG1). Microalgae were treated with UV-B (1.7 W m^−2^) for 3 days prior to their exposure to CBW and then incubated for 4 days in the absence or presence of UV-B. UV-B-untreated microalgae were used as the control. Comparative physiological responses, including photosynthetic pigments and non-enzymatic antioxidants, as well as nicotine and nicotyrine removal, were evaluated in 7-day cultures. UV-B treatments did not negatively impact algal chlorophyll or carotenoid production. UV-B acclimation was strain-dependent, correlating with native environment adaptations and genetic constitutions. UV-B as a pretreatment had long-term positive effects on non-enzymatic antioxidant capacity. However, LG1 needed more time to readjust the pro-oxidant/antioxidant balance, as it was the most UV-B-sensitive. Phenolic compounds played an important role in the antioxidant system response to UV-B, while flavonoids did not contribute to the total antioxidant capacity. Although cross-resistance between UV-B and CBW was observed in F4 and R1, only R1 showed nicotine/nicotyrine catabolism induction due to UV-B. Overall, the results suggest that UV-B activates defense pathways associated with resistance or tolerance to nicotine and nicotyrine.

## 1. Introduction

Cigarette butts (CBs) are the main type of massive waste, which can be found in urban zones, oceans, forests and even protected areas due to their improper disposal [1]. When it occurs, several toxic compounds (e.g., heavy metals, polycyclic aromatic hydrocarbons, N-nitrosamines, aromatic amines, benzene and nicotine) leach into the environment, affecting terrestrial and aquatic organisms to different degrees, ranging from alterations in behavior to physiological/developmental abnormalities and death [2,3,4,5,6]. Surprisingly, despite their toxicity, CBs are considered “Municipal waste including separately collected fraction/Separately collected fraction/Other fractions not otherwise specified” at the regulatory level [7]. Moreover, conventional disposal systems for CBs (e.g., landfilling and incineration) are unsustainable and release hazardous contaminants to the environment; thus, an effective management solution for this waste is essential to significantly reduce environmental repercussions [8].

On the basis of the circular economy and sustainable development, previous studies proposed a novel solution to recycle the filters of smoked cigarettes into soilless substrates, where CBs were first cleaned up, and then the wastewater was treated with microalgae [9,10]. In fact, the remediation of wastewater using microalgae has been employed to remove a variety of pollutants, including organic and inorganic compounds, which can be effectively converted into valuable products, such as biomass, following the principle of “waste-to-wealth” [11]. However, there are several challenges to overcome in order to maximize the advantages of this technology. For instance, changes in cultivation parameters, such as light, can affect microalgal growth, which in turn influences the capacity to remove contaminants in wastewater. In fact, light is the primary source of energy for the growth and development of photosynthetic organisms, including microalgae.

Ultraviolet (UV) radiation represents a small fraction of the sunlight spectrum, and only wavelengths greater than 290 nm (UV-B and UV-A) reach the Earth’s surface due to the stratospheric ozone layer [12]. The UV-B effects on living organisms have received increasing scientific attention over the past three decades because of the detection of ozone layer depletion and the concomitant increase in harmful UV-B [13,14]. In plants, UV-B drives a mix of damaging and acclimation responses by inducing UV-B-specific and/or -nonspecific signaling pathways [15,16,17]. Besides terrestrial impacts, UV-B can also penetrate the water column to a considerable depth, reaching freshwater and marine organisms [18]. In algae, UV-B can alter their physiological and biochemical activities to different extents depending on the algal species, UV-B intensity and exposure time [19]. Recently, the UV-B photoreceptor UVR8 (UV RESISTANCE LOCUS 8), which was first characterized in *Arabidopsis thaliana*, was identified in green algae, bryophytes, lycophytes and angiosperms [20,21,22]. Moreover, Zhang et al. [23] identified orthologous genes of the core UVR8 signaling module in green algae, indicating that the mechanism of action is well-conserved with a chlorophytic origin. At a biotechnological level, the use of UV-B, as well as other abiotic stresses, has been strategically applied as a tool in the microalgal machinery of biorefineries in order to enhance the biosynthesis of high-value products, such as antioxidants, pigments (carotenoids) and lipids (omega-3 polyunsaturated fatty acids) [24]. However, exploring the use of UV-B as a possible stimulator of antioxidant activities in microalgae to counteract wastewater pollutants has not yet been investigated.

It was found in a previous study that nicotine and nicotyrine were difficult to remove by microalgae during CB wastewater treatment, especially at high concentrations (25% CB) [10], implying that more efficient approaches are needed to totally remove these pollutants. Therefore, in this study, an evaluation was performed on the antioxidant responses of *Chlorella sorokiniana* to short acute UV-B irradiation and their remediation capacity for nicotine and nicotyrine contained in CB wastewater. The tested hypothesis was that UV-B would induce antioxidant activity in some microalgal strains, which, in turn, would contribute to counteracting the oxidative stress triggered by toxic agents in the wastewater during bioremediation. To this end, cultures of three *C. sorokiniana* strains isolated from native conditions, named F4, R1 and LG1 [10,25], were exposed to UV-B before CB wastewater treatment (as a sequential experiment) or before and during CB wastewater treatment (as a parallel experiment). Thus, the aim of this work was to compare the physiological responses of the *C. sorokiniana* strains, including photosynthetic pigments and non-enzymatic antioxidant activities, as well as their removal of nicotine and nicotyrine in both sequential and parallel experiments in order to evaluate the hypothesis. Here, this paper presents the first study that reports the use of UV-B as a tool to strengthen antioxidant responses in microalgae in order to remove nicotine and nicotyrine contained in CB wastewater for its remediation.

## 2. Results

### 2.1. Photosynthetic Pigments in Microalgal Strains

**Figure 1:**
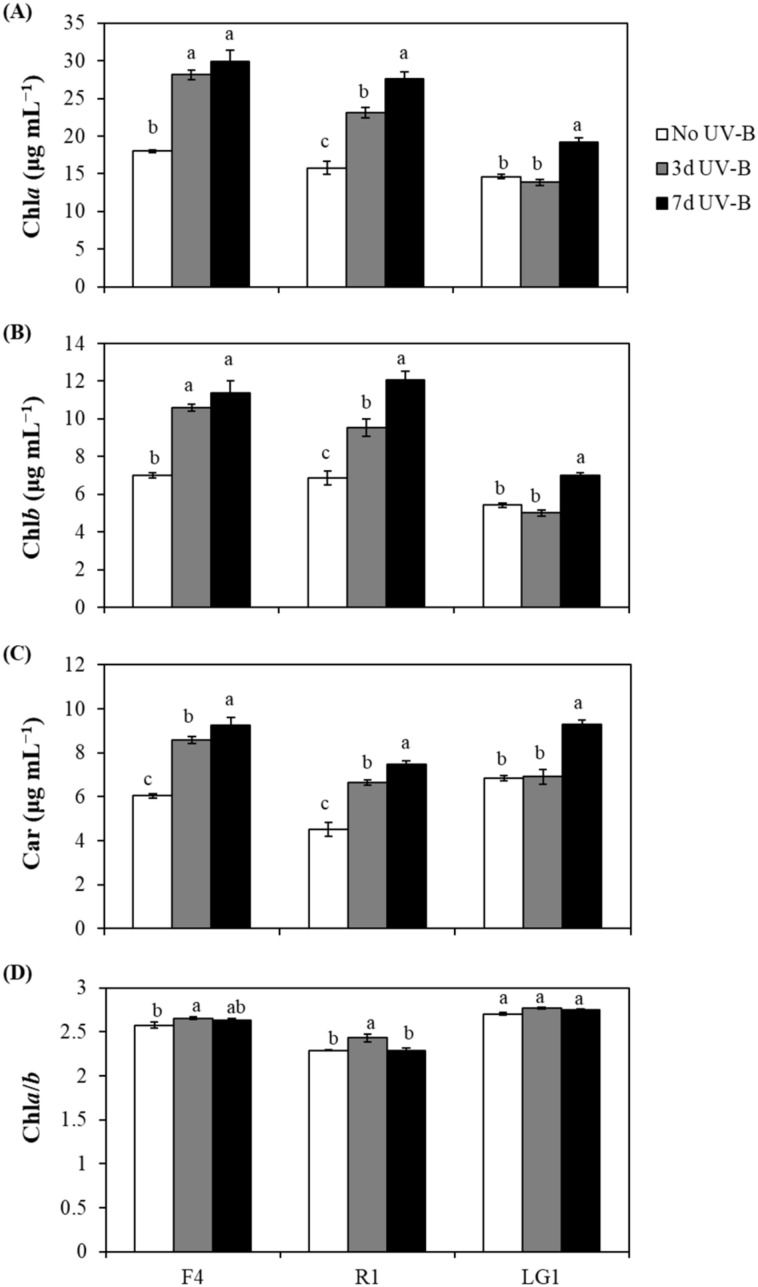
Effect of UV-B on photosynthetic pigments in microalgae subjected to cigarette butt (CB)-derived wastewater treatment. (**A**) Chlorophyll *a* (Chl*a*), (**B**) chlorophyll *b* (Chl*b*), (**C**) carotenoids (Car) and (**D**) the ratio of Chl*a* to Chl*b* (Chl*a*/*b*) were determined in each microalgal strain (F4, R1 and LG1) in 7-day cultures. Microalgae were exposed to wastewater and sequential (3 d UV-B) or parallel (7 d UV-B) UV-B treatments. The UV-B exposure level was set at 1.7 W m^−2^ and supplemented with 70 μmol m^−1^ s^−1^ photosynthetically active radiation (PAR) for 20 min each day. The control microalgal group only received PAR (No UV-B). For more details, see Section 4. Different letters represent significant differences (*p* < 0.05) between UV-B treatments within the same strain. Data are expressed as means of 4 different replicates ± standard error (SE).

UV-B radiation generally increased the contents of photosynthetic pigments in microalgae subjected to CB wastewater treatment, with some differences between UV-B exposure periods depending on the strain (Figure 1A–C). In detail, chlorophyll *a* (Chl*a*) and chlorophyll *b* (Chl*b*) in F4 did not show significant differences between 3 and 7 d UV-B-exposed cells, while in R1, they were significantly higher when cells were exposed to 7 d UV-B (Figure 1A,B). Concerning carotenoids (Car), F4 and R1 showed significantly higher contents in cells exposed to 7 d UV-B than in those exposed to 3 d UV-B (Figure 1C). Conversely, in LG1, the induction of photosynthetic pigments due to UV-B radiation was only observed when cells were exposed to 7 d UV-B, while no significant differences were detected between No UV-B and 3 d UV-B-treated cells (Figure 1A–C). Concerning the ratio between Chl*a* and Chl*b* (Chl*a*/*b*), LG1 subjected to CB wastewater treatment did not show significant differences between No UV-B and UV-B-treated cells (Figure 1D). Conversely, Chl*a*/*b* in F4 and R1 did not show significant differences between No UV-B and 7 d UV-B-treated cells, while a significant increase was observed when cells were exposed to 3 d UV-B (Figure 1D).

### 2.2. Non-Enzymatic Antioxidants in Microalgal Strains

**Figure 2:**
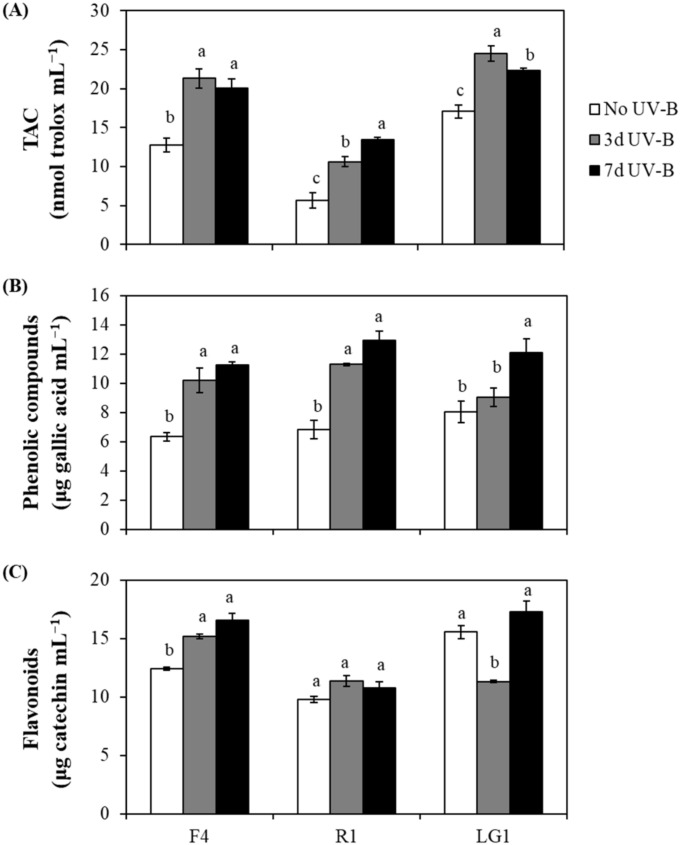
Effect of UV-B the non-enzymatic antioxidants in microalgae subjected to cigarette butt (CB)-derived wastewater treatment. (**A**) Total antioxidant capacity (TAC), (**B**) phenolic compounds and (**C**) flavonoids were determined in each microalgal strain (F4, R1 and LG1) in 7-day cultures. Microalgae were exposed to wastewater and sequential (3 d UV-B) or parallel (7 d UV-B) UV-B treatments. The UV-B exposure level was set at 1.7 W m^−2^ and supplemented with 70 μmol m^−1^ s^−1^ photosynthetically active radiation (PAR) for 20 min each day. The control microalgal group only received PAR (No UV-B). For more details, see Section 4. Different letters represent significant differences (*p* < 0.05) between UV-B treatments within the same strain. Data are expressed as means of 4 different replicates ± standard error (SE).

In general, the total antioxidant capacity (TAC) in all strains subjected to CB wastewater treatment was induced by UV-B radiation, with some differences between UV-B exposure periods depending on the strain (Figure 2A). TAC in F4 did not show significant differences between 3 and 7 d UV-B treatments, while in R1, it was significantly increased with UV-B exposure, and in LG1, it was significantly reduced (Figure 2A). The phenolic compounds in F4 and R1 were induced by UV-B without differences between exposure periods (Figure 2B). However, LG1 only showed a significant increase in phenolic compounds when cells were exposed to 7 d UV-B, while no significant differences were detected between No UV-B and 3 d UV-B-treated cells (Figure 2B). Concerning flavonoids, F4 showed an increase with UV-B without significant differences between exposure periods, while R1 did not show significant differences between No UV-B and UV-B-treated cells (Figure 2C). In contrast, flavonoids in LG1 did not show significant differences between No UV-B and 7 d UV-B-treated cells, while a significant decrease was observed when cells were exposed to 3 d UV-B (Figure 2C).

### 2.3. CB Wastewater Subjected to Microalgal-Based Remediation

**Figure 3:**
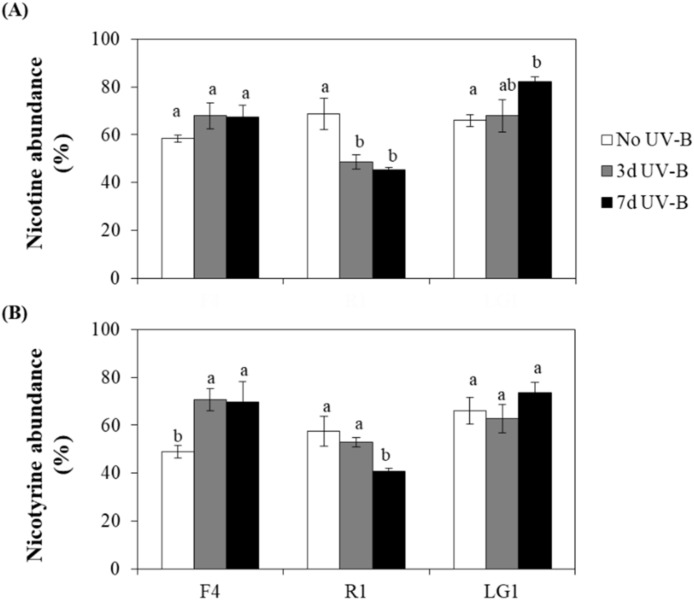
Effect of UV-B on the microalgal removal capacity of main toxic pollutants in cigarette butt (CB) wastewater. The relative abundance of (**A**) nicotine and (**B**) nicotyrine was determined in the wastewater after microalgal-based remediation by 7-day cultures. The abundance is expressed in % (kcounts/kcounts) and was obtained by its comparison with the abundance in the respective untreated wastewater (without microalgal cells), which represented 100%. Microalgal strains (F4, R1 and LG1) were exposed to wastewater and sequential (3 d UV-B) or parallel (7 d UV-B) UV-B treatments. The UV-B exposure level was set at 1.7 W m^−2^ and supplemented with 70 μmol m^−1^ s^−1^ photosynthetically active radiation (PAR) for 20 min each day. The control microalgal group only received PAR (No UV-B). For more details, see Section 4. Different letters represent significant differences (*p* < 0.05) between UV-B treatments within the same strain. Data are expressed as means of 4 different replicates ± standard error (SE).

This study confirmed that nicotine and nicotyrine were the most difficult organic compounds to remove by microalgae when exposed to 25% CB wastewater, as previously reported [10]. Nicotine abundance in the wastewater after microalgal-based remediation was, on average, approximately 65% when strains grew in the absence of UV-B (No UV-B). However, the remediation capacity was significantly affected by UV-B in the strains R1 and LG1, with no effects in F4 (Figure 3A). In R1, UV-B increased the remediation capacity, and approximately 47% of nicotine was detected in the wastewater without significant differences between 3 and 7 d UV-B (Figure 3A). On the other hand, the removal capacity of nicotine in LG1 was compromised with increasing UV-B exposure (Figure 3A). Concerning nicotyrine, no UV-B effect was observed on the remediation capacity of LG1, as nicotyrine levels in the wastewater did not show significant differences between No UV-B and UV-B-treated cells (Figure 3B). In F4, UV-B decreased the remediation capacity without significant differences between 3 and 7 d UV-B, while the removal capacity of nicotyrine in R1 was significantly increased only when cells were exposed to 7 d UV-B (Figure 3B).

### 2.4. Multiple Factor Analysis

**Figure 4:**
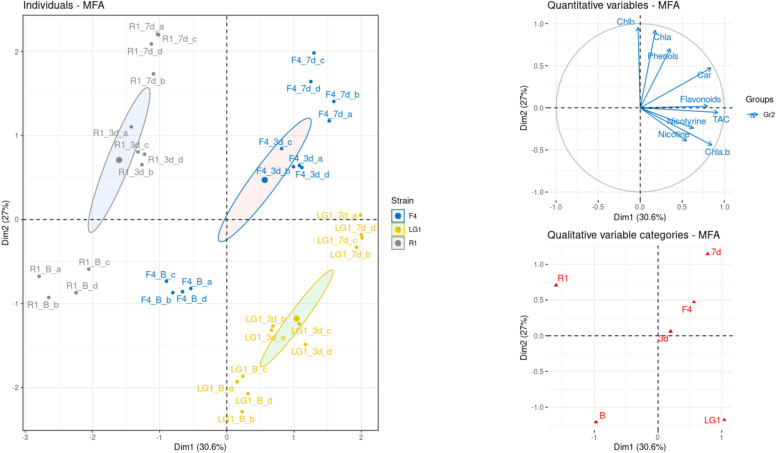
Multiple factor analysis (MFA) of physiological and analytical data on microalgal-based remediation of wastewater from a cigarette butt (CB) cleaning process, coupled with UV-B treatment. B: No UV-B irradiation; 3d: 3-day UV-B treatment; 7d: 7-day UV-B treatment; Chla.b: ratio of Chl*a* to Chl*b*; a, b, c and d indicate replicates.

The multiple factor analysis (MFA) shown in Figure 4 revealed not only the distinct separation of the three microalgal strains but also the separation of each treatment within each microalgal strain dataset (No UV-B, 3 d UV-B and 7 d UV-B). For this purpose, and according to the qualitative variables, the treatments (i.e., exposure time to UV-B) had a strong effect, shaping the plot along the y-axis. Observing the quantitative variables, strains R1 and F4 were strongly related to the vector of the Chl*a* and Chl*b* content, especially in the 7 d UV-B treatment and slightly in the 3 d UV-B and No UV-B treatments. At the same time, they also seemed to be unrelated to the high contents of nicotine and nicotyrine, as the vector has the opposite direction. On the other side, strain LG1 seemed to be negatively affected by the treatments and related to the highest contents of nicotine and nicotyrine (No UV-B and 3 d UV-B treatments) and to the highest values of TAC and flavonoids (7 d UV-B treatment).

## 3. Discussion

At the biotechnological level, abiotic stresses such as UV-B have been strategically applied as a tool in the microalgal machinery of biorefineries in order to enhance the biosynthesis of high-value products such as pigments, lipids and polymers [24]. However, exploring the use of UV-B in microalgal-based wastewater remediation has not yet been studied. Here, we present the first study that reports the use of UV-B as a tool to strengthen antioxidant responses in microalgae to remove nicotine and nicotyrine contained in CB wastewater for its remediation.

A previous study demonstrated that the production of photosynthetic pigments of microalgae was strongly inhibited by 25% CB, highlighting the toxicity of CB wastewater pollutants [10]. Conversely, in this study, the pigments of microalgae exposed to CB wastewater were generally increased upon UV-B exposure, indicating that UV-B can mitigate and/or prime *C. sorokiniana* cells against CB wastewater pollutants. Moreover, Chl*a*/*b* showed slight changes due to UV-B, indicating a negligible effect on light-harvesting complexes, in contrast to the distress effect that usually causes strong changes in the Chl*a*/*b* ratio [26,27,28,29]. The UV-B effect on microalgal growth was also strain-dependent, which may be related to their adaptation to their native environments. In fact, F4 and R1 were isolated from an inland swamp (Fucecchio Marshland, Italy) and from a water sample from a private terrace (Pisa, Italy), respectively, while LG1 was isolated from a plant substrate in a growth chamber [10,25]. Thus, it is plausible that UV-B acted as “positive stress” in F4 and R1, as they had naturally grown in the presence of ambient UV-B instead of in its absence, like LG1.

There is a general consensus that UV-B exerts an overall deleterious effect on the photosynthetic apparatus. For instance, enhanced UV-B exposure to microalgae resulted in a decrease in photosynthetic pigments and yield, which was intensified with exposure time [30]. However, it has been demonstrated that many photosynthetic organisms, including microalgae, are also able to acclimate to UV-B by adjusting their metabolism and preventing damage [22,26]. In this study, when F4 and R1 received UV-B as a pretreatment followed by CB wastewater exposure (i.e., 3d UV-B), UV-B had a long-term positive effect on photosynthetic pigments. Previous studies showed that pigment content in *Dunaliella salina* was induced by short-term UV-B irradiation at different doses, and this effect was probably regulated by photomorphogenic photoreceptors [23,31,32]. Thus, it is possible that the UV-B pretreatment in this study enhanced photoprotection pathways in F4 and R1, similar to plants [33], that overlapped with defense pathways against CB wastewater pollutants. This cross-resistance effect due to UV-B was also observed in LG1 but only when UV-B and CB wastewater were applied in the parallel treatment (i.e., 7d UV-B), suggesting that the applied UV-B dose may stimulate acclimation responses in long periods, as this strain had never been exposed to UV-B in its native environment. Another interesting aspect of the present study was that R1 was the only strain that potentially showed a synergistic effect on cross-resistance when UV-B and CB wastewater were applied in the parallel treatment (i.e., 7 d UV-B), as photosynthetic pigments were significantly higher than those in No UV-B and 3 d UV-B treatments. Surprisingly, F4 showed this possible synergistic effect only on Car under parallel UV-B and CB wastewater treatment, highlighting the promising photoprotective function of carotenoids, probably related to their high antioxidant activity [19,34], which overlaps with the deactivation of reactive oxygen species (ROS) induced by the wastewater [35]. However, further studies are necessary to determine what type of carotenoid is involved in this cross-resistance between UV-B and wastewater pollutants.

Many abiotic and biotic variables trigger the production of ROS, which can pose a threat to cells but also act as signals for the activation of antioxidants [36]. Hao et al. [35] demonstrated that pollutants in wastewater derived from the tobacco industry induce ROS accumulation in *C. pyrenoidosa*, resulting in cellular damage and impairing microalgal growth. This growth impairment was also observed in *C. sorokiniana* exposed to CB wastewater [10], indicating that CB wastewater pollutants may induce ROS production in microalgal cells. Concerning UV-B, it can be a potential source of oxidative stress; however, depending on the dose and energy, this abiotic variable can be less detrimental, as induced ROS may play an important role in UV-B acclimation and metabolism readjustment [37]. For instance, single high acute UV-B (4.6 W m^−2^) irradiation doses in *C. vulgaris* induced ROS formation, resulting in serious photooxidative damage when the irradiation time was increased (63 vs. 155 min) [38], while low UV-B (0.7 W m^−2^) irradiation for 4 days allowed UV-B acclimation and tolerance in *Chlamydomonas reinhardtii* [22]. The alteration between pro-oxidants and scavenging activity in response to UV-B also depends on the species or even the genotype of the same species [26]. For instance, *C. vulgaris* and *Chlorella* sp. may develop different antioxidant activities in response to high acute UV-B (4.6 W m^−2^) irradiation related to different native environment adaptations and genetic constitutions to cope with UV-B [39]. In this study, the total capacity of non-enzymatic antioxidants (TAC) in all strains had a positive long-term effect in response to UV-B pretreatment followed by CB wastewater exposure (i.e., 3d UV-B), suggesting that UV-B might initiate a series of defense pathways that provide increased protection against toxic pollutants in the CB wastewater. Interestingly, TAC showed different patterns in the parallel treatment (i.e., 7d UV-B) depending on the strain, highlighting their different capacities of UV-B acclimation, probably linked to their native environments. Concordantly, the MFA showed that LG1 correlated to the highest values of TAC, indicating that this strain was more sensitive to the applied UV-B dose than the other strains and needed an acclimation period to readjust the balance between pro-oxidants and antioxidants. Phenolic compounds in this study showed similar patterns to TAC, underlining their important role in the antioxidant system in response to UV-B [40,41]. Although green algae such as *Chlorella* contain plant-specific UV-B photoreceptor orthologs, no conservation was found in the downstream component related to flavonoid biosynthesis (i.e., *MYB13* transcription factor) [23,42]. In this study, a contribution of flavonoids to total antioxidant capacity was not found, indicating that other photoprotectants were more relevant during UV-B acclimation [14]. Moreover, the involvement of antioxidant enzymes in the crosstalk between UV-B and CB pollutants is not excluded, as enzymatic and non-enzymatic antioxidants may have a good complementary ability in antioxidation [41].

Overall, the results suggest that UV-B stimuli might activate defense pathways associated with the resistance or tolerance to CB wastewater by the neutralization of ROS production, highlighting the importance of crosstalk between the two abiotic variables, with promising biotechnological applications, especially under indoor conditions. Furthermore, it is well known that crosstalk is governed by a complex interaction between signaling pathways, resulting in outcomes such as cross-resistance, cross-tolerance or cross-sensitivity, which may vary depending on the species or even the genotype of the same species [43]. For instance, changes in two abiotic stresses, such as nitrogen source and temperature, resulted in high lipid accumulation for biodiesel production depending on the microalgal species [44]. In this study, among the three *C. sorokiniana* strains, R1 was the only one in which UV-B seemed to have a synergistic effect on cross-resistance to the most persistent and toxic CB pollutants (i.e., nicotine and nicotyrine), where UV-B-exposed cells may not only gain protection against pollutants (ROS-induced cellular damage) but also improve the catabolic pathways involved in the removal of these alkaloids. Further large-scale studies are needed to validate the process at the pre-industrial level.

## 4. Materials and Methods

### 4.1. CB Collection and Cleaning Process

The collection and treatment (100 g L^−1^) of cigarette butts (CBs) were performed as previously described by Chiellini et al. [10]. Briefly, CBs from public collectors in different coffee bars near the municipality of Capannori (Lucca, Italy) were cleaned by exhaust boiling in distilled water for 10 min. This cleaning process was performed in quadruplicate, and the individual wastewaters were treated with different microalgae.

### 4.2. Microalgal Strains and Growth Conditions

Three *Chlorella sorokiniana* strains, namely, F4, R1 and LG1, were previously isolated and characterized [10,25]. All strains were grown in sterile tris-acetate-phosphate (TAP) medium in a growth chamber at a controlled temperature (23 ± 1 °C) with a 16/08 h light/dark cycle and 70 μmol m^−1^ s^−1^ photosynthetically active radiation (PAR). The aforementioned culture conditions were also maintained throughout the UV-B and wastewater treatment.

### 4.3. UV-B Radiation Treatment

**Figure 5:**
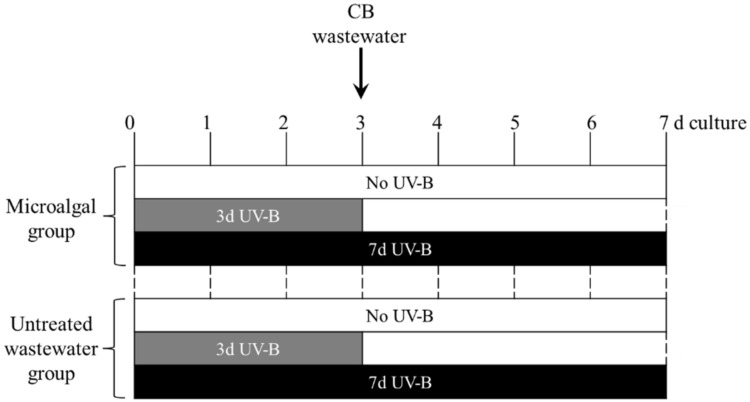
Experimental design of the study. All treatments were carried out under PAR. UV-B irradiation was applied either before (3 d UV-B) or in parallel to (7 d UV-B) a 4-day wastewater treatment. The control microalgal group only received PAR (No UV-B). Another parallel untreated wastewater group (no microalgae) was included, and it was exposed to each UV-B treatment (No UVB, 3 d UV-B and 7 d UV-B). Sampling was performed in 7-day cultures. See Section 4.2 and Section 4.3 for details of treatment conditions. CB; cigarette butt.

A volume of 200 µL of microalgal culture was added to 24-well plates (1.5 cm diameter, Greiner Bio-one, Kremsmünster, Austria) containing 1300 µL of fresh TAP medium per well. UV-B radiation was applied using a Philips TL 20W/01RS UV-B Narrowband lamp (Koninklijke Philips Electronics, Eindhoven, The Netherlands) with a peak emission at 311 nm. The intensity of UV-B was determined by adjusting the distance between the UV-B lamp and multiwell plates and measured using a UV-B meter (Skye Instruments Ltd., Powys, UK). The UV-B exposure level was set at 1.7 W m^−2^ and supplemented with 70 μmol m^−1^ s^−1^ PAR for 20 min each day (at 12:00). UV-B treatment was performed in two different experiments: (i) applied for 3 days before wastewater treatment as a sequential experiment (3 d UV-B) or (ii) applied for 7 days, including the 4-day wastewater treatment, as a parallel experiment (7d UV-B). In addition, a parallel microalgal group was kept in the absence of UV-B (No UV-B) and used as a control (Figure 5). Similarly, another parallel untreated wastewater group without microalgal cells (1500 µL TAP per well) was included, and it was exposed to each UV-B treatment (No UVB, 3 d UV-B and 7 d UV-B) (Figure 5).

### 4.4. Evaluation of Microalgal Remediation

After 3-day culture with or without UV-B, 500 µL of filter-sterilized CB wastewater (0.45 µm cellulose acetate filter, Sartorius, Göttingen, Germany) was added to each well to obtain a 25% (*v*/*v*) wastewater dilution (Figure 5). At the end of the experiment, cultures in each well were centrifuged at 3000× *g* for 10 min, and the supernatant and the microalgal pellet were collected separately for further analysis.

### 4.5. Extraction and Determination of Photosynthetic Pigments

Photosynthetic pigments, including chlorophyll *a* (Chl*a*), chlorophyll *b* (Chl*b*) and total carotenoids (Car), were extracted from microalgal pellets and analyzed as previously reported [45]. Four biological replicates were considered for these analyses.

### 4.6. Extraction and Determination of Total Antioxidant Capacity, Phenolic Compounds and Flavonoids

Extraction was performed as described by Moles et al. [46] with some modifications. Briefly, microalgal pellets were extracted in 80% ethanol. Samples were sonicated (Branson 1210 sonicator, Bransonic, Connecticut, USA) for 30 min at room temperature, incubated for another 30 min in the dark and then centrifuged at 10,000 rpm for 10 min. The ethanolic extracts were recovered and then used for determining the total antioxidant capacity (TAC), phenolic compounds and flavonoids. TAC was spectrophotometrically determined at 515 nm by the 2,2-diphenyl-1-picrylhydrazyl (DPPH) assay, as reported by Huarancca Reyes et al. [47]. Phenolic compounds were assayed with the method based on Folin–Ciocalteau reagent and spectrophotometrically determined at 750 nm, as described by Huarancca Reyes et al. [16]. Total flavonoids were spectrophotometrically determined at 510 nm, referring to Mariotti et al. [48]. Four biological replicates were considered for these analyses.

### 4.7. Analytical Determinations

Supernatants were dried under vacuum and diluted with acetone and heptane 50% (*v*/*v*). Nicotine [pyridine, 3-(1-methyl-2-pyrrolidinyl)] and nicotyrine [pyridine, 3-(1-methyl-1H-pyrrol-2-yl)] in the wastewater samples were determined as described by Chiellini et al. [10]. The quantification was performed using the relative abundance of their chromatogram peaks (instrument detection limit < 400 counts). The abundance of nicotine (retention time = 6.63 min) and nicotyrine (retention time = 8.577 min) in the wastewater after microalgal-based remediation was expressed in % (kcounts/kcounts) and obtained by its comparison with the abundance in the respective untreated wastewater, which represented 100%. Four biological replicates were considered for these analyses.

### 4.8. Statistical Analyses

The values presented are means of four replicates. Statistical analysis was performed using one-way analysis of variance (ANOVA). Tukey’s test was used to determine significant differences among means (*p* < 0.05). All computations were performed with the software STATISTICA for Windows version 14.0 (Stat-Soft, Inc., Tulsa, OK, USA).

To identify the effect of UV-B irradiation for the improvement of the remediation capacity of microalgae toward nicotine- and nicotyrine-containing CB wastewater, based on physiological and analytical data, multiple factor analysis (MFA) was carried out [49]. Data were normalized using Z-score calculation, and the R software [50] packages “FactoMineR” (analysis) and “factoextra” (visualization) were applied.

## Data Availability

Data are contained within the article.
